# Decreasing birth weight is associated with adverse metabolic profile and lower stature in childhood and adolescence

**DOI:** 10.1186/1687-9856-2015-S1-P110

**Published:** 2015-04-28

**Authors:** José Derraik, Deborah Rowe, Wayne Cutfield, Paul Hofman

**Affiliations:** 1 Liggins Institute, University of Auckland, New Zealand

## Objective

We aimed to evaluate the association of birth weight SDS (BWSDS) with insulin resistance, blood pressure, and auxology in children and adolescents born 23–42 weeks of gestation.

## Methods

We studied 143 singleton children and adolescents aged 9.3 ± 3.3 years (range 2.0 – 17.9 years). Clinical assessments included auxology, insulin resistance measured by the HOMA2-IR, and blood pressure from sphygmomanometer measurements. Continuous associations were examined and stratified analyses carried out. For the latter, participants were divided into those of below-average birth weight (BBW, <0 SDS) and above-average birth weight (ABW, ≥0 SDS).

## Results

Irrespective of gestational age, lower BWSDS was associated with progressively increased insulin resistance (p<0.0001) and fasting insulin concentrations (p<0.0001). Decreasing BWSDS was associated with higher systolic (p=0.011) and diastolic (p=0.006) blood pressure. Lower BWSDS was also associated with decreasing stature (p<0.010). The BBW group was ~40% more insulin resistant than ABW participants (p=0.004), with the former also displaying fasting insulin concentrations 37% higher (p=0.004). BBW participants were 0.34 SDS shorter than those of higher birth weight (p=0.002). On average, BBW participants failed to meet their genetic potential, tending to be shorter than their parents (p=0.065). As a result, when corrected for parents' heights, BBW participants were 0.6 SDS shorter than those born of higher birth weight (p=0.001). Sub-group analyses on participants born appropriate-for-gestational-age (n=128) showed that associations of BWSDS with both insulin resistance and stature remained (although attenuated).

## Conclusion

Decreasing BWSDS (even within the normal range) is associated with adverse metabolic profile and lower stature in children and adolescents.

